# Physical fitness mediates and predicts for high blood pressure among children in relation to weight status

**DOI:** 10.3389/fpubh.2023.1157351

**Published:** 2023-04-18

**Authors:** Hai-Hua Chuang, Wen-Jin Cherng, Chih-Hung Lin, Li-Ang Lee, Kuang-Hung Hsu, Rong-Ho Lin

**Affiliations:** ^1^Department of Family Medicine, Taipei and Linkou Main Branches, Chang Gung Memorial Hospital, Taoyuan, Taiwan; ^2^Metabolism and Obesity Institute, Taipei and Linkou Main Branches, Chang Gung Memorial Hospital, Taoyuan, Taiwan; ^3^Department of Industrial Engineering and Management, National Taipei University of Technology, Taipei, Taiwan; ^4^College of Medicine, Chang Gung University, Taoyuan, Taiwan; ^5^School of Medicine, National Tsing Hua University, Hsinchu, Taiwan; ^6^Division of Cardiology, Department of Internal Medicine, Chang-Gung Memorial Hospital, Linkou Main Branch, Taoyuan, Taiwan; ^7^Department of Plastic and Reconstructive Surgery, Linkou Main Branch, Chang-Gung Memorial Hospital, Taoyuan, Taiwan; ^8^Departments of Otorhinolaryngology-Head and Neck Surgery, Linkou Main Branch, Chang-Gung Memorial Hospital, Taoyuan, Taiwan; ^9^Healthy Aging Research Center, Chang Gung University, Taoyuan, Taiwan; ^10^Laboratory for Epidemiology, Department of Health Care Management, Chang Gung University, Taoyuan, Taiwan; ^11^Department of Emergency Medicine, Chang Gung Memorial Hospital at Linkou, Taoyuan, Taiwan

**Keywords:** blood pressure, body mass index, childhood obesity, pediatric hypertension, physical fitness, standing long jump

## Abstract

**Background:**

Pediatric hypertension contributes to adulthood hypertension and target organ damage. Obesity is a well-known predictor for pediatric hypertension; however, the relationship between physical fitness and blood pressure (BP) is unclear among children. This study aimed to compare the differences in demographics, anthropometrics, and physical fitness across BP subgroups and investigate whether physical fitness was related to pediatric hypertension independent of weight status.

**Methods:**

This quantitative, cross-sectional study investigated demographic, anthropometric, physical fitness, and BP measures among 360 healthy school-aged children. Continuous variables were compared across BP subgroups with the one-way analysis of variance. Mediation and moderation analyses were used to explore the mechanism. Multivariable regression models were used to assess independent associations for hypertension.

**Results:**

There were 177 (49.2%), 37 (10.3%), and 146 (40.6%) children in the normotensive, elevated BP, and hypertensive subgroups, respectively. The hypertensive subgroup had higher body mass index (BMI) and waist/height ratio percentiles and performed worse in 800-m run, standing long jump (SLJ), and 1-min sit-ups than the normotensive subgroup. Furthermore, the 800-m run percentile (total effect: β = 0.308, standard error = 0.044, *p* < 0.001) and sit and reach percentile (total effect: β = 0.308, standard error = 0.044, *p* < 0.001) mediated the relationship between the BMI percentile and systolic BP percentile; the SLJ percentile was directly associated with the diastolic BP percentile (β,−0.197, 95% confidence interval,−0.298−0.097; *p* < 0.001). The parsimonious model of multivariable regression models revealed that the SLJ percentile (adjusted exp (β), 0.992, 95% confidence interval, 0.985–0.999; *p* = 0.042) and BMI percentile (adjusted exp (β), 1.024, 95% confidence interval, 1.016–1.032; *p* < 0.001) were two independent predictors for pediatric hypertension.

**Conclusion:**

Physical fitness mediates the relationship between anthropometric and BP measures. The SLJ percentile is associated with pediatric hypertension independent of the BMI percentile. Proactive screening and health promotion for not only healthy weight status but also good physical fitness may be beneficial for BP control among school-aged students.

## 1. Introduction

Hypertension is well-documented as a risk factor for cardiovascular diseases (1), chronic kidney disease (2), and premature mortality (3). A meta-analysis in 2019 revealed that the global prevalence of pre-hypertension and hypertension was 9.67% and 4.00% among children 19 years and younger (4). Without effective management, pediatric hypertension is likely to develop into adulthood hypertension (5) and eventually lead to target organ damage (6). However, early identification and intervention of hypertension remain to be a challenge, especially among the pediatric population (7). Compared to adulthood hypertension, pediatric hypertension is diagnosed less promptly and controlled inadequately (7, 8). Although ambulatory BP monitoring is more accurate for diagnosing hypertension than clinic-measured BP (9), in most clinical settings, it is not easy to be implemented routinely for children and adolescents (10). Therefore, identifying at-risk individuals is vital for the timely diagnosis and management of pediatric hypertension (11).

Known risk factors for primary hypertension in children include male sex, older age, overweight and obesity, high sodium intake, physical inactivity, and obstructive sleep apnea (12–14). Some evidence has indicated associations between BP and anthropometric measures. Lo et al. found that waist-to-height ratio (WHtR) had significant screening power for metabolic syndrome components in children, including hypertension (15). Wang et al. revealed that abdominal skin fold was more closely associated with hypertension than body mass index (BMI) in Chinese boys and girls (16). Moreover, previous high-quality epidemiological studies supported the reverse association of physical activity with adolescent/pediatric hypertension (17–19).

While physical activity is defined as any bodily movement produced by skeletal muscles that result in energy expenditure, physical fitness is a set of attributes that are either health- or skill-related and can be measured only with specific tests. Physical fitness represents a general health status to perform daily life functions in work and leisure activities (20). The major health-related components of physical fitness include body composition, cardiorespiratory fitness, musculoskeletal fitness, and motor fitness (21). Physical fitness is a powerful marker of health regarding abdominal adiposity, cardiovascular disease risk factors, skeletal health, quality of life, mood status, and academic performance in children and adolescents (22). In an earlier study of ours, we discovered that body composition parameters predicted pediatric hypertension better than conventional anthropometric measures (23). Some other studies have also reported a connection between physical fitness and BP in children. For example, low cardiorespiratory fitness was related to elevated systolic BP (SBP) in children (24–26). Furthermore, change in physical fitness was inversely associated with the change in BP in school-aged children (27), and persistently low muscle strength contributed to subsequently raised levels of both SBP and diastolic BP (DBP) in adolescents (28). However, the results of studies on the associations between physical fitness and pediatric hypertension have been inconclusive after adjustment for body weight status (29–31).

We hypothesized that poor physical fitness was a risk factor for high blood pressure in children. The study aimed to compare the differences in demographic, anthropometric, and physical fitness measurements across BP subgroups, to explore the mediating and moderating roles of physical fitness on the relationship between body weight status and BP status, and to investigate whether physical fitness was independently associated with pediatric hypertension after adjusting for body weight status in a sample of school-aged children in Taiwan.

## 2. Materials and methods

### 2.1. Participants

This cross-sectional study utilized a quantitative analysis to evaluate anonymous data gathered from a multidisciplinary health promotion program, administered by Chang Gung Memorial Hospital's Linkou Main Branch between 2013 and 2016. The program's target audience consisted of 1,860 students, aged between 6 and 13 years, from four elementary schools in Guishan District—a suburban city located close to Taipei in Northern Taiwan—with a population of nearly 160,000. The parents of the students in this cohort were predominantly employed in the electronics, manufacturing, and healthcare industries (32). The Institutional Review Board of the Chang Gung Medical Foundation, Taoyuan, Taiwan (No. 101-4158A3) approved the program, and written informed consent was obtained from all participants and their parents. Details regarding the program have been previously described (32). This study adhered to the principles outlined in the World Medical Association Declaration of Helsinki (33).

We enrolled seemingly healthy fourth- to sixth-grade children to investigate whether physical fitness is a risk factor for high blood pressure, while also accounting for conventional risk factors such as male sex, older age, and overweight/obesity. These grades were chosen due to the availability of normative reference data on physical fitness. The inclusion criteria were as follows: (1) age ranged from 9 to 12 years; and (2) complete demographic, anthropometric, physical fitness, and BP data. The exclusion criteria were as follows: (1) a history of high BP or under treatment for high BP; and (2) any history of chronic illness such as diabetes mellitus, asthma, chronic pain, cystic fibrosis, congenital heart disease, attention-deficit/hyperactivity disorder, or depression (34, 35). Demographic (sex and age), anthropometric, physical fitness, and BP data were retrieved for statistical analysis.

### 2.2. Anthropometric measures

Body mass index (BMI) (kg/m^2^) was defined as body weight (kg) divided by the body height squared (m^2^). BMI percentiles were calculated based on sex and age in months according to the United States Centers for Disease Control and Prevention 2,000 growth charts (36). Waist circumference (in cm) was measured in the horizontal plane midway between the lowest ribs and the iliac crest. WHtR was calculated as waist circumference (cm) divided by the body height (cm) (37), and WHtR percentiles were obtained based on sex and age in years according to the United States National Health and Nutrition Survey, cycle III (38).

### 2.3. Physical fitness

In this study, the levels of physical fitness were measured by four exercise assessments, including 800-m run (800 mR), standing long jump (SLJ), 1-min sit-ups (1 mSU), and sit and reach (S&R). All physical fitness measurements were performed following the guidelines of the Ministry of Education in Taiwan (39). The detailed protocol of physical fitness measurements had been reported previously (40). Professional physical coaches conducted the assessments and recorded the results. The test results were expressed in percentiles based on sex and age in years according to the reference values for physical fitness of students aged 7–23 years in Taiwan (41).

The 800 mR (s) was defined as the time required for a participant to sprint an 800-m run (42). The 800 mR represented cardiorespiratory fitness and endurance (43).

The SLJ (cm) was defined as the maximum distance between the starting line and the heel of the closest foot after a participant took off for a forward and upward jump and landed on both feet (44). The SLJ represented lower body (leg muscles) strength (45).

The 1 mSU (times) was defined as the maximum number of correct sit-ups achieved within 1 min (46). The participant lay on a mat with knees bent, and arms crossed upon their chest. When the participant touched their knees with their elbows, it was considered a count. 1 mSU represented abdominal muscle strength and endurance (47).

The S&R (cm) was defined by the most distant point reached on the ruler with the fingertips. The participants slide their hands forward as far as possible toward their feet without bending the hamstring and maintaining the top position for at least 2 s (48). Each participant performed three times; the longest measurement out of three was used. The S&R represented the flexibility of the hamstrings (49).

### 2.4. Blood pressure measurements and categories

After the participant sat for at least 10 min in the classroom, SBP and DBP were measured using an automated sphygmomanometer. If a child's BP exceeded the normal range, the investigator rechecked SBP and DBP after a 5-min rest. BP was measured two times, and the lowest SBP and DBP-values were used (23).

The SBP percentile and DBP percentile were obtained based on age in years, sex, and height z-score according to the BP reference tables published in 2017 (50) as an update to the 2004 “Fourth Report on the Diagnosis, Evaluation, and Treatment of High Blood Pressure in Children and Adolescents” (10). Normal BP was defined as BP of <90th percentile; elevated BP was defined as SBP and/or DBP of ≥90th to <95th percentile or 120/80 mmHg to <95th percentile (whichever is lower); pediatric hypertension was defined as SBP and/or DBP of ≥95th percentile (50).

### 2.5. Statistical analysis

The stratum sample size was calculated at 32 based on Pearson's correlation between cardiorespiratory fitness and systolic blood pressure (*r* = 0.43) (24) under a type-I error of 0.05 and statistical power of 0.95. Considering the heterogeneity of relationships across strata (three different grade groups, two sexes, and three blood pressure groups), a minimum of 324 children were required. Therefore, we included a representative cohort of 360 children.

Most continuous variables were normally distributed using the Kolmogorov–Smirnov test. Therefore, continuous variables were reported as means ± standard deviations (SD), and categorical variables were summarized as numbers (percentages). Furthermore, continuous variables were compared using the one-way analysis of variance with *post hoc* Tukey's honestly significant difference tests, and categorical variables were compared using the Mantel–Haenszel test for trend among three BP subgroups. The curve estimation procedure was used to identify useful functional relationships, and Pearson's correlation test was used to assess relationships between continuous variables.

All variables were included in the full models using multivariable linear or binary logistic regression models as appropriate. To identify independent variables of the parsimonious model, all variables were initially included; the variable with the highest but insignificant *p*-value of ≥ 0.05 was removed at one step, and the manual selection procedures were repeated until all variables with a *p*-value of < 0.05. To adjust the intervariable relationship within the model, the variance inflation factor of each variable was calculated and removed if its value was ≥ 5 to reduce the multicollinearity (51). Furthermore, conditional process analyses were performed to evaluate the mediation and moderation of selected variables using a PROCESS macro for SPSS (version 4.1) (52). Using 5,000 runs of bootstrapping, bias-corrected 95% confidence intervals (CIs) were estimated to verify mediation, moderated mediation, or mediated moderation. A *p*-value of < 0.05 was considered to be statistically significant. Statistical analysis was performed using SPSS software version 25.0 (International Business Machines Corp., Armonk, NY, USA).

## 3. Results

### 3.1. Participants characteristics

From a database of 1,860 elementary-school students, 1,066 were in grades 4 to 6. Among them, 533 have complete data of selected variables; 67 were excluded due to a history of high BP or under treatment for high BP, and 106 were excluded due to chronic illness. Therefore, a total of 360 children (180 [50.0%] girls and 180 [50.0%] boys) with a mean age of 10.0 ± 0.8 years (range, 9–12 years) were included in the study. There were 177 (49.2%), 37 (10.3%), and 146 (40.6%) children in the normotensive, elevated BP, and hypertensive subgroups, respectively. Furthermore, 96 (26.7%) children had isolated systolic hypertension, 22 (6.1%) children had isolated diastolic hypertension, and 28 (7.8%) had simultaneous systolic and diastolic hypertension.

### 3.2. Differences in participants' characteristics across various BP subgroups

Table 1 summarizes the demographic, anthropometric, physical fitness, and BP measures in the overall cohort and in the normotensive, elevated BP, and hypertensive subgroups. For anthropometrics, the hypertensive subgroup had the highest BMI percentile and WHtR percentile among all three subgroups. For physical fitness, the hypertensive subgroup had worse outcomes of the 800 mR percentile, SLJ percentile, and 1 mSU percentile compared to the normotensive subgroup. In addition, BP measures (including the SBP percentile and DBP percentile) of the hypertensive subgroup were higher than those of the normotensive and elevated BP subgroups, even though the distributions of age and sex were comparable across the three subgroups.

**Table 1:** Demographic, anthropometric, physical fitness, and blood pressure measures of the overall cohort as well as normotensive, elevated blood pressure, and hypertensive subgroups.

**Variables Patients**	**Overall *n* = 360**	**Normotensive subgroup *n* = 177**	**Elevated blood pressure subgroup *n* = 37**	**Hypertensive subgroup *n* = 146**	***p*-value^a^**
**Demographic measures**					
Girls (*n*)	180 (50.0)	89 (50.3)	17 (45.9)	74 (50.7)	0.956
Age (years)	10.0 (0.8)	10.1 (0.8)	10.0 (0.9)	10.0 (0.9)	0.586
**Anthropometric measures**					
BMI (kg/m^2^)	18.8 (3.9)	17.4 (3.1)^b^	18.2 (3.0)^c^	20.5 (4.4)^b^^,^^c^	< 0.001
BMI percentile (%)	60.3 (31.8)	49.5 (30.5)^b^	60.4 (32.2)^c^	73.3 (28.2)^b^^,^^c^	< 0.001
WHtR	0.46 (0.06)	0.44 (0.05)^b^	0.45 (0.05)^c^	0.49 (0.07)^b^^,^^c^	< 0.001
WHtR percentile (%)	47.0 (32.3)	37.4 (29.6)^b^	44.6 (30.0)^c^	59.3 (32.1)^b^^,^^c^	< 0.001
**Physical fitness parameters**					
800 mR (s)	306.9 (70.3)	298.2 (61.5)^b^	291.4 (51.2)^c^	321.8 (81.5)^b^^,^^c^	0.004
800 mR percentile (%)	51.0 (27.6)	53.9 (26.2)^b^	57.4 (23.8)	45.9 (29.4)^b^	0.012
SLJ (cm)	137.1 (25.9)	141.1 (25.8)^b^	137.6 (26.7)	132.1 (25.2)^b^	0.008
SLJ percentile (%)	57.5 (30.5)	61.2 (29.8)^b^	55.8 (33.8)	51.8 (29.6)^b^	0.006
1 mSU (times)	27.8 (8.9)	28.8 (9.0)^b^	29.3 (9.1)	26.2 (8.7)^b^	0.018
1 mSU percentile (%)	58.9 (27.6)	62.5 (27.0)^b^	62.7 (25.6)	53.7 (28.1)^b^	0.011
S&R (cm)	27.8 (8.5)	25.5 (8.1)	25.8 (11.0)	25.8 (8.5)	0.944
S&R percentile (%)	45.7 (27.2)	45.3 (26.6)	47.9 (32.7)	45.6 (26.6)	0.866
**Blood pressure measures**					
SBP (mmHg)	111.7 (14.5)	101.3 (7.7)^b^	110.4 (9.0)^b^^,^^c^	124.5 (11.4)^b^^,^^c^	< 0.001
SBP percentile (%)	68.3 (28.9)	53.8 (24.6)^b^	81.2 (24.1)^b^^,^^c^	94.5 (12.2)^b^^,^^c^	< 0.001
DBP (mmHg)	63.1 (12.2)	56.9 (8.4)^b^	63.3 (9.7)^b^^,^^c^	70.5 (12.6)^b^^,^^c^	< 0.001
DBP percentile (%)	53.3 (29.3)	39.1 (24.8)^b^	58.0 (27.4)^b^^,^^c^	71.5 (27.0)^b^^,^^c^	< 0.001

Data summarized as mean (standard deviation) or *n* (%) as appropriate.

a Data were compared using one-way analysis of variance with *post hoc* Tukey's honestly significant difference tests or Mantel–Haenszel test for trend as appropriate.

b *p*-Value < 0.05 when the variable in the normotensive subgroup was compared with the elevated blood pressure or hypertensive subgroup.

c *p*-Value < 0.05 when the variable in the elevated blood pressure subgroup was compared with the hypertensive subgroup.

BMI, body mass index; DBP, diastolic blood pressure; SBP, systolic blood pressure; S&R, sit and reach; SLJ, standing long jump; WHtR, waist/height ratio; 1 mSU, 1-min sit and ups; 800 mR, 800-m run.

### 3.3. Relationship between study variables

Table 2 demonstrates the associations of BP measures with physical fitness and anthropometric variables in the overall cohort. Higher SBP percentile was related to higher DBP, BMI, and WHtR percentiles, as well as lower 800 mR and 1 mSU percentiles. After adjustment for the BMI and WHtR percentiles, the SBP percentile was significantly associated with the 800 mR percentile (*r* = −0.12; *p* = 0.025) and S&R percentile (*r* = 0.14; *p* = 0.009). A higher DBP percentile was related to higher BMI and WHtR percentiles and lower SLJ and 1 mSU percentiles. After adjustment for the BMI and WHtR percentiles, the DBP percentile was significantly associated with the SLJ percentile (*r* = −0.18; *p* < 0.001) and 1 mSU percentile (*r* = −0.13; *p* = 0.017). A higher BMI percentile was associated with a higher WHtR percentile and lower 800 mR, SLJ, 1 mSU, and S&R percentiles. A higher WHtR percentile was associated with lower 800 mR, SLJ, and 1 mSU percentiles. A higher 800 mR percentile was associated with higher SLJ and 1 mSU percentiles. A higher SLJ percentile was associated with higher 1 mSU and S&R percentiles. A higher 1 mSU percentile was associated with a higher S&R percentile.

**Table 2:** Pearson correlations of blood pressure measures with anthropometric and physical fitness measures in the overall cohort.

**Variables**	**SBP percentile**	**DBP percentile**	**BMI percentile**	**WHtR percentile**	**800 mR percentile**	**SLJ percentile**	**1 mSU percentile**	**S&R percentile**
SBP percentile	–							
DBP percentile	0.38^c^	–						
BMI percentile	0.35^c^	0.13^a^	–					
WHtR percentile	0.36^c^	0.11^a^	0.76^c^	–				
800 mR percentile	−0.22^c^	−0.08	−0.26^c^	−0.29^c^	–			
SLJ percentile	−0.08	−0.20^c^	−0.15^b^	−0.19^c^	0.27^c^	–		
1 mSU percentile	−0.15^b^	−0.14^b^	−0.16^b^	−0.25^c^	0.44^c^	0.36^c^	–	
S&R percentile	0.10	−0.02	−0.11^a^	−0.09	0.06	0.31^c^	0.12^a^	–

Data are summarized as r-value (*p*-value). The significant level is indicated as follows:

a *p*-Value ≥ 0.01– < 0.05,

b *p*-Value ≥ 0.001– < 0.01,

c *p*-Value < 0.001.

### 3.4. Independent associations between physical fitness, weight status, and blood pressure

Table 3 summarizes independent associations of the BP percentile with anthropometric and physical fitness measures in the overall cohort. Multivariable linear regression modules showed BMI, WHtR, and S&R percentiles were significantly positively associated with the SBP percentile in the full model. Using manual selection approaches, BMI, WHtR, and S&R percentiles were positively related to the SBP percentile, whereas the 800 mR percentile was inversely related to the SBP percentile in the parsimonious model. The SLJ percentile was independently inversely related to the DBP percentile in the full model and the parsimonious model.

**Table 3:** Independent associations of blood pressure measures with anthropometric and physical fitness measures in the overall cohort.

**Variables**	**Full model**					**Parsimonious model**		
	β	**95% CI**	***p*** **-Value** ^a^	**VIF**	β	**95% CI**	***p*** **-Value** ^b^	**VIF**
**SBP percentile**								
BMI percentile	0.167	0.037–0.296	0.012	2.363	0.162	0.033–0.291	0.014	2.347
WHtR percentile	0.161	0.032–0.291	0.015	2.445	0.171	0.044–0.299	0.009	2.385
800 mR percentile	−0.098	−0.209–0.013	0.083	1.319	−0.120	−0.222–0.019	0.020	1.098
SLJ percentile	−0.021	−0.120–0.078	0.675	1.285			Not significant	
1 mSU percentile	−0.043	−0.156–0.071	0.461	1.367			Not significant	
S&R percentile	0.146	0.043–0.250	0.006	1.113	0.136	0.037–0.231	0.007	1.013
	*R*^2^ = 0.173				*R*^2^ = 0.171			
**DBP percentile**								
BMI percentile	0.115	−0.033–0.263	0.127	2.363			Not significant	
WHtR percentile	−0.025	−0.173–0.123	0.742	2.444			Not significant	
800 mR percentile	0.031	−0.096–0.159	0.628	1.322			Not significant	
SLJ percentile	−0.177	−0.261–0.063	0.002	1.282	−0.197	−0.298−0.097	< 0.001	1.000
1 mSU percentile	−0.093	−0.223–0.036	0.158	1.364			Not significant	
S&R percentile	0.058	−0.060–0.177	0.335	1.110			Not significant	
	*R*^2^ = 0.058				*R*^2^ = 0.040			

a All anthropometric and physical fitness variables were included for multivariable linear regression models.

b All anthropometric and physical fitness variables were included for multivariable linear regression models with manual selections.

CI, confidence interval; VIF, variance inflation factor.

### 3.5. Mediation and moderation analysis

**Figure 1:**
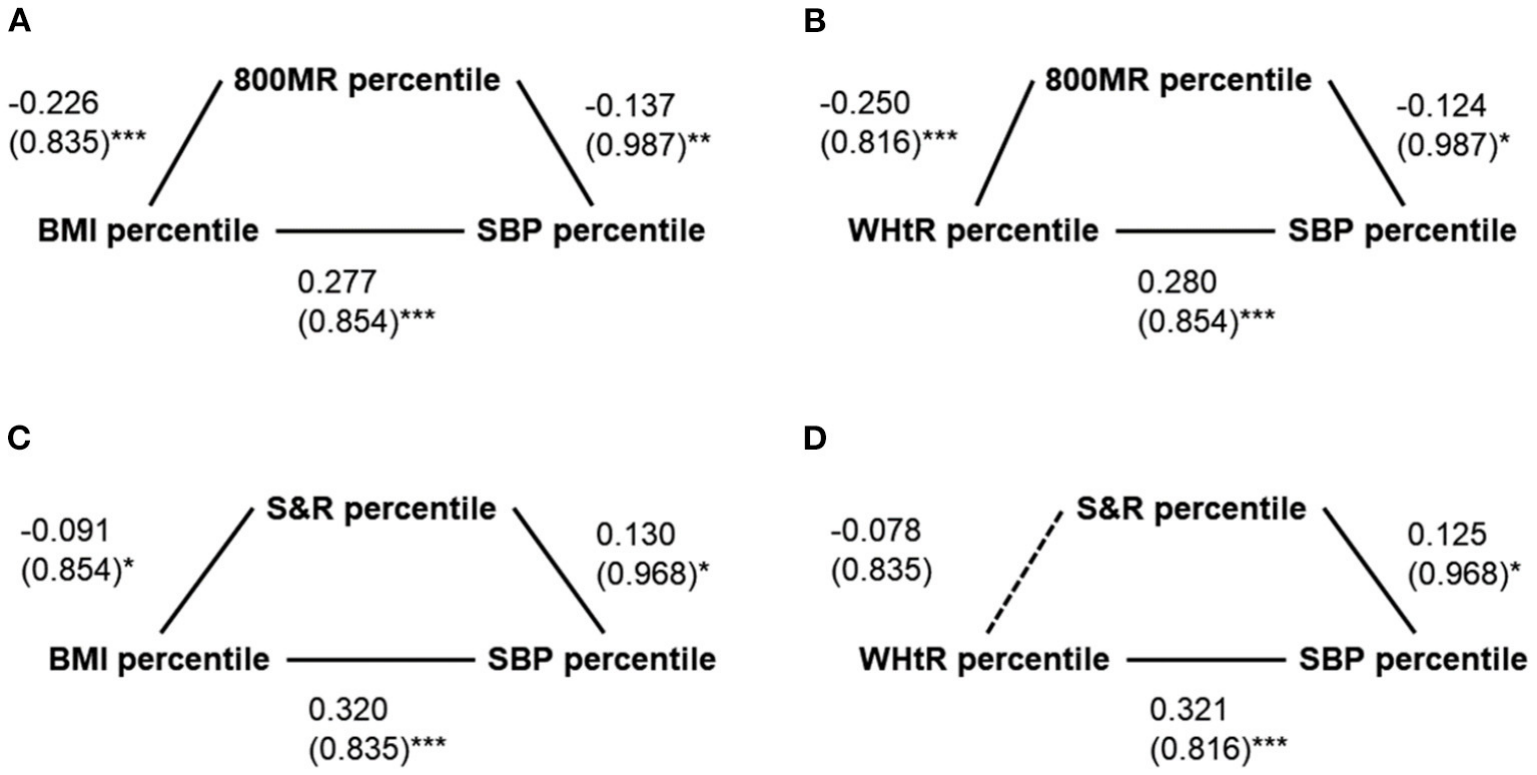
Simple mediation models of possible mediators of the relationships between anthropometric and blood pressure measures. **(A)** The 800-m run (800 mR) percentile significantly mediated the relationship between the body mass index (BMI) percentile and the systolic blood pressure (SBP) percentile. **(B)** The 800 mR percentile significantly mediated the relationship between the waist/height ratio (WHtR) percentile and the SBP percentile. **(C)** The S&R significantly mediated the relationship between the BMI percentile and the SBP percentile. **(D)** The S&R percentile did not significantly mediate the relationship between the WHtR percentile and the SBP percentile. Data are summarized as β (standard deviations). ^*^p < 0.05 and ≥ 0.01; ^**^p < 0.01 and ≥ 0.001; ^***^p < 0.001.

Since there were nested data structures and significant correlations among BP, anthropometric, and physical fitness measures, we performed mediation and moderation analyses to investigate the role of physical fitness measures on the relationship between anthropometric and BP measures. The main findings included the following:

1. The 800 mR percentile significantly mediated the relationship between the BMI percentile and SBP percentile (total effect = 0.308, SD = 0.835, 95% CI: 0.222–0.394, *p* < 0.001; direct effect = 0.277, SD = 0.854, 95% CI: 0.189–0.365, *p* < 0.001; indirect effect = 0.031, SD = 0.247, 95% CI: 0.010–0.066; mediation percentage = 10.1%) (Figure 1A) as well as the relationship between the WHtR percentile and SBP percentile (total effect = 0.311, SD = 0.816, 95% CI: 0.227–0.395, *p* < 0.001; direct effect = 0.280, SD = 0.854, 95% CI: 0.192–0.368, *p* < 0.001; indirect effect = 0.031, SD = 0.266, 95% CI: 0.006–0.059; mediation percentage = 10.0%) (Figure 1B).
2. The S&R percentile significantly mediated the relationship between the BMI percentile and SBP percentile (total effect = 0.308, SD = 0.835, 95% CI: 0.222–0.394, *p* < 0.001; direct effect = 0.320, SD = 0.835, 95% CI: 0.234–0.406, *p* < 0.001; indirect effect = −0.012, SD = 0.133, 95% CI: −0.032−0.0002; mediation percentage = −3.9%) (Figure 1C); however, the S&R percentile did not mediate the relationship between the WHtR percentile and SBP percentile (total effect = 0.311, SD = 0.816, 95% CI: 0.227–0.395, *p* < 0.001; direct effect = 0.321, SD = 0.816, 95% CI: 0.237–0.405, *p* < 0.001; indirect effect = −0.010, SD = 0.133, 95% CI: −0.025–0.002; mediation percentage = −3.2%) (Figure 1D).

### 3.6. Independent predictors for pediatric hypertension

Table 4 summarizes independent predictors for pediatric hypertension in the overall cohort. Using multivariable binary logistic regression models, a higher BMI percentile was significantly correlated with pediatric hypertension in the full model. Using manual selection approaches, a higher BMI percentile and a lower SLJ percentile were associated with pediatric hypertension in the parsimonious model. A higher BMI percentile and a higher S&R percentile were significantly related to systolic hypertension in the full models; however, a higher BMI percentile and lower 800 mR were independently associated with systolic hypertension in the parsimonious model. A lower SLJ percentile was correlated with diastolic hypertension in the full model; furthermore, a higher BMI percentile and a lower SLJ percentile were independently related to diastolic hypertension in the parsimonious model.

**Table 4:** Independent predictors for pediatric hypertension with anthropometric and physical fitness measures in the overall cohort.

**Variables**	**Full model**					**Parsimonious model**		
	**Exp(**β**)**	**95% CI**	***p*** **-value** ^a^	**VIF**	**Exp(**β**)**	**95% CI**	***p*** **-value** ^b^	**VIF**
**Hypertension**								
BMI percentile	1.019	1.007–1.030	0.001	2.363	1.024	1.016–1.032	< 0.001	1.024
WHtR percentile	1.006	0.995–1.017	0.271	2.445			Not significant	
800 mR percentile	0.999	0.990–1.009	0.909	1.319			Not significant	
SLJ percentile	0.993	0.984–1.001	0.090	1.285	0.992	0.985–0.999	0.042	1.024
1 mSU percentile	0.995	0.985–1.004	0.266	1.367			Not significant	
S&R percentile	1.006	0.997–1.015	0.168	1.113			Not significant	
	*R*^2^ = 0.187				*R*^2^ = 0.170			
**Systolic hypertension**								
BMI percentile	1.018	1.006–1.031	0.003	2.363	1.023	1.015–1.032	< 0.001	1.072
WHtR percentile	1.007	0.996–1.018	0.229	2.445			Not significant	
800 mR percentile	0.993	0.984–1.003	0.174	1.319	0.990	0.982–0.999	0.031	1.072
SLJ percentile	0.997	0.988–1.005	0.462	1.285			Not significant	
1 mSU percentile	0.996	0.986–1.006	0.456	1.367			Not significant	
S&R percentile	1.010	1.001–1.019	0.038	1.113			Not significant	
	*R*^2^ = 0.192				*R*^2^ = 0.169			
**Diastolic hypertension**								
BMI percentile	1.015	0.998–1.031	0.077	2.363	1.012	1.001–1.023	0.029	1.023
WHtR percentile	0.996	0.981–1.011	0.626	2.445			Not significant	
800 mR percentile	1.003	0.990–1.016	0.658	1.319			Not significant	
SLJ percentile	0.984	0.972–0.995	0.006	1.285	0.982	0.972–0.992	0.001	1.023
1 mSU percentile	0.995	0.982–1.007	0.396	1.367			Not significant	
S&R percentile	0.997	0.985–1.009	0.626	1.113			Not significant	
	*R*^2^ = 0.104				*R*^2^ = 0.098			

a All anthropometric and physical fitness variables were included for multivariable binary logistic regression models.

b All anthropometric and physical fitness variables were included for multivariable binary logistic regression models with manual selections.

## 4. Discussion

Obesity is one of the most critical risk factors for pediatric hypertension. A substantial volume of research has supported the roles of anthropometric measures (such as BMI, WHtR, and waist circumference) and body composition parameters (such as fat mass and fat-free mass) in predicting pediatric hypertension (23, 53, 54). In line with the literature, our data showed that the hypertensive subgroup had higher BMI and WHtR percentiles; also, higher BMI and WHtR percentiles were related to the SBP percentile independent of physical fitness variables. Along with growth and development, weight status may be quite varying during childhood. The Clinical Practice Guideline developed by the European Society of Endocrinology and the Pediatric Endocrine Society has suggested regular evaluation, early identification, and prompt intervention to prevent the detrimental effects of pediatric obesity (55).

However, the connections between physical fitness and BP in children are not as conclusive as those between weight status and BP. Some research has shown that better physical fitness is associated with lower diastolic and systolic BP in children and adolescents despite mechanisms not yet fully understood (28, 30, 56, 57). Dong et al. used a complex and summative physical fitness indicator composed of forced vital capacity, SLJ, 800 mR, S&R, and 1 mSU, which could significantly predict raised systolic and diastolic hypertension in children aged 7–18 years; however, S&R was not related to high BP (26). Nevertheless, physical fitness did not well predict BP in children aged 6–11 years (58). Furthermore, a meta-analysis of randomized controlled trials conducted by García-Hermoso et al. in 2020 showed that physical exercise interventions improved BMI, waist circumference, and physical fitness, but not BP in preschoolers (59). These findings indicate that the relationships and interactions between physical fitness and pediatric hypertension need to be further delineated to yield more precise prevention and intervention strategies in terms of health promotion.

Current evidence suggests that cardiorespiratory fitness is overall inversely related to BP and multiple cardiovascular risk factors despite some discordances between studies with different epidemiological, methodological, and analytical approaches. Diaz et al. demonstrated that better cardiorespiratory fitness was associated with decreased probability of BP elevation, lower insulin resistance, and improved liver and renal functions (28, 30, 56, 57). Köchli et al. reported a less favorable micro- and macro-vascular profile in children aged 6–8 years with poor cardiorespiratory fitness (60). De Moraes et al. found that low cardiorespiratory fitness and muscular fitness accurately predicted high BP in children aged 3–17 years (61). Furthermore, Ayala-Guzmán et al. found that the relationship between low cardiorespiratory fitness and hypertension was not significant after correlation with BMI in children aged 9 to 12 years (29). The current study supported the linkage between poor cardiorespiratory fitness and high BP in children aged 9–12 years, and we further revealed that the impact was mainly on systolic BP, independent of weight status. Moreover, cardiorespiratory fitness mediated the relationships between the BMI percentile and WHtR percentile with the SBP percentile (Figures 1A, B). Future research is warranted to define clinically relevant cutoff points of cardiorespiratory fitness (62, 63) for children. Moreover, the effectiveness of cardiorespiratory fitness promotion on health outcomes needs to be examined from a public health perspective (29).

Notably, according to our data, the lower SLJ percentile was an independent and critical physical fitness indicator in predicting both hypertension and diastolic hypertension, even after adjustment for anthropometric variables. The SLJ percentile neither mediated nor moderated the associations of the DBP percentile with the BMI percentile or WHtR percentile; the effect of SLJ performance on DBP was direct. The SLJ, also known as the standing long jump, requires pushing the total body mass forward and is one of the most common physical fitness tests for children to assess lower body muscle strength. The SLJ is highly associated with isokinetic measures of lower extremity force (64). The calf muscles, including gastrocnemius and soleus, are involved in the movements of an SLJ. It is called “the second heart” by some as it pumps blood back to the trunk and improves circulation during walking and exercising (65). Calf muscle pump dysfunction can result in venous hypertension (66). Agostinis-Sobrinho found that a low level of muscular fitness was associated with a high inflammatory status in adolescents (62, 63). Delgado Floody et al. demonstrated that low maximal oxygen consumption and low body muscle strength were positively associated with high SBP (67) in children aged 11–13 years.

The solo and inverse association between the SLJ percentile and DBP percentile was a novel and particularly interesting finding of this study. In contrast to older people, in whom isolated diastolic hypertension is less prevalent and not associated with CV outcomes, emerging evidence suggests that the linkage between isolated diastolic hypertension and adverse CV effects is particularly significant in younger individuals and requires treatment (68). Cohen et al. reported a similar finding to the current study: handgrip had a protective effect against BP elevation, especially DBP (28, 30, 56, 57). Moreover, in a Korean multidisciplinary lifestyle intervention program, children and adolescents with moderate-to-severe obesity significantly improved their weight status, body composition, and DBP, but not SBP (69, 70). Future investigations on the pathogenesis of diastolic hypertension among the youth and its linkage to muscular fitness will be of interest.

Literature has suggested that the adverse effects of low physical fitness are likely to be prevented or even reversed by increasing physical activity. For example, an 8-week fitness course in indoor cycling can improve BMI, waist circumference, physical fitness (lower body muscle strength and aerobic fitness), and BP (71). Limiting sedentary behaviors, proper nutrition, increasing physical activity, and resting sufficiently may promote physical fitness and health outcomes (72, 73). Exercise can increase physical fitness and reduce BP in children and adolescents (69, 70) *via* the activation of adaptive mechanisms to improve endothelial function, induce pro-angiogenic pathways, and increase insulin sensitivity (74). Furthermore, engaging in moderate-to-vigorous physical activity, such as aerobic exercise, to achieve an improved (75) or a within-recommended level of cardiorespiratory fitness (76) reduces the incidence of hypertension. The results of the current study again highlight the importance of the assessment and promotion of physical fitness among children.

The present study has remarkable strengths derived from its comprehensive evaluation of physical fitness and BP with the exemplary sample size and the heterogeneous participants, recruited from different schools in northern Taiwan. However, this study has several limitations which merit discussion. First, all the participants were school students in Taiwan and mainly Han. The mean intake of sodium by Taiwanese children was significantly higher than the Daily Reference Intake of Taiwan or other recommended standards (77), furthermore, children in Guishan had the highest prevalence of overweight and obesity compared to nearby areas (78), therefore the cohort of this study was at risk of high BP. This may limit the generalizability of the study. Second, the BP measurements were not standardized for medical diagnosis of hypertension as there was no confirmation through auscultation or ambulatory BP monitoring. Therefore, conditions such as white coat hypertension, masked hypertension, or isolated nocturnal hypertension could not be identified (79). Instead, automated BP measurement with the lowest SBP and DBP readings was adopted. Third, there are many exercise tests to assess the same physical fitness component [for example, 20-m aerobic cardiovascular endurance run test (25, 29), 6-min running/walking test (30), maximal ergometer cycle test (24) to cardiorespiratory fitness, or push-ups (29), and manual dynamometer (31) to measure muscular endurance], construct validity may differ across various exercise tests. Fourth, this cross-sectional study could not conclude the causality of physical fitness-mediated hypertension in children and the results should be interpreted cautiously. Longitudinal case-control and interventional studies are warranted to confirm the role of lower body muscle strength in pediatric hypertension.

In conclusion, the present study demonstrated that suboptimal weight status and physical fitness were two major risk factors for high BP among children. The BMI percentile was independently associated with the SBP percentile, systolic hypertension, and pediatric hypertension. The 800 mR was independently associated with the SBP percentile and systolic hypertension; it also mediated the relationship between weight status and BP. The SLJ was independently associated with the DBP percentile, diastolic hypertension, and pediatric hypertension. Not only weight management but also physical fitness promotion is vital for the pediatric population and the focus should be on both cardiorespiratory and muscular fitness.

## Data availability statement

The original contributions presented in the study are included in the article/supplementary material, further inquiries can be directed to the corresponding author.

## Ethics statement

The studies involving human participants were reviewed and approved by Institutional Review Board of the Chang Gung Medical Foundation, Taoyuan, Taiwan. Written informed consent to participate in this study was provided by the participants' legal guardian/next of kin.

## Author contributions

H-HC, W-JC, K-HH, and R-HL conceived and planned the study. H-HC enrolled the patients. H-HC, W-JC, L-AL, and R-HL designed the study, analyzed data, made the statistics, and interpreted the results. H-HC, W-JC, L-AL, K-HH, and R-HL participated in manuscript drafting. C-HL, K-HH, and R-HL supervised the study. All authors read and approved the final manuscript.
